# Supporting general practices to develop green action plans to reduce carbon emissions: development and evaluation of the feasibility of a workshop-based intervention

**DOI:** 10.1017/S1463423626101145

**Published:** 2026-03-27

**Authors:** Olivia Geddes, Helen Twohig, Abi Eccles, Helen Atherton, Frederik Dahlmann, Florence Karaba, Ana Raquel Nunes, Rachel Spencer, Nicky Thomas, Jeremy Dale

**Affiliations:** 1 University of Warwick Medical School, UK; 2 Keele University School of Medicine, UK; 3 University of Oxford Nuffield Department of Primary Care Health Sciences, UK; 4 University of Southampton Faculty of Medicine Health and Life Sciences: University, UK; 5 Warwick Business School: University of Warwick Business School, UK

**Keywords:** Climate change, decarbonization, educational video, general practice

## Abstract

**Aim:**

To describe the development, delivery, and outcome of an action-oriented intervention comprising an awareness-raising educational video and workshop designed to support general practice teams to identify and plan decarbonization actions, delivered from May-September 2024.

**Background:**

Healthcare services internationally are committing to net zero targets. General practice is recognized as having a pivotal role in achieving these ambitions. However, limited awareness of decarbonization initiatives and insufficient support for implementation highlight the need for an educational resource to facilitate action planning.

**Methods:**

Principles of organizational change, video-design, and barriers to decarbonization informed the intervention’s development. The video included modules featuring resource materials and ideas to support the development and implementation of decarbonization actions in general practice. Prompts for a facilitated workshop discussion were developed to support action planning. The intervention was delivered to 64 multidisciplinary staff across 12 general practices in England. A conceptual content analysis was conducted on completed practice green action plans (GAPs) and data from an online participant feedback form were analysed using descriptive statistics to assess perceptions of the intervention. Free-text comments were thematically analysed.

**Results:**

Across the 12 GAPs, each practice planned between three and eight decarbonization actions. ‘Managing waste’ was the most frequently addressed area, appearing in 10 practice GAPs, and most planned actions mapped onto those presented within the video. Thirty (46.9%) participants completed the evaluation survey. The intervention was well received, with 28 (93.3%) survey respondents rating the overall usefulness of the video as 4 or 5 (1 ‘not at all useful’ to 5 ‘very useful’). Free-text comments for suggested improvements related to time for consolidating learning, and concerns about the video’s audio quality and duration.

**Conclusions:**

The educational workshop successfully facilitated the development of structured GAPs with explicit timescales and intended outcomes. This study did not assess the implementation of planned actions.

## Introduction

### Background

The health effects of climate change are well understood (IPCC, 2023; UK Health Security Agency, 2023; Romanello et al., 2024;), and the resulting pressure on healthcare systems threatens to undermine the past 50 years of public health gains (Tennisson et al., 2021). Health systems, not only crucial in the response to climate change, also contribute significantly to the crisis (Tennisson et al., 2021). Acknowledging this, the United Kingdom’s (UK) National Health Service (NHS), alongside other health systems across the globe, have committed to net zero targets (NHS, 2020; WHO, 2021). The NHS strives to reach net zero by 2040 for the emissions it controls directly, with an 80% reduction between 2028–2032, and by 2045 for those it can influence (NHS, 2020). General practice is recognized as being pivotal in achieving this ambition. However, despite a multitude of resource materials available, practical guidance on how to effectively support and implement decarbonization in this setting is limited, even though it has a significant emissions footprint (Tennisson et al., 2021; BMA, 2023).

Previous research has identified several clinical, and non-clinical opportunities for decarbonization in general practice (Karaba et al., 2024; Larsen et al., 2025; Nunes et al., 2025), examples including interventions around asthma inhaler prescribing (Woodcock et al., 2022; Franklin et al., 2025), and energy and waste reduction (Fehrer et al., 2023; Pavli et al., 2023; Hale and McKenzie, 2024). Furthermore, there are a burgeoning array of resource materials available to support general practice teams to decarbonize, including e-learning modules (RCGP., 2023), social networks (Greener Practice, 2017), practical toolkits (Green Impact, 2015), and a non-clinical carbon calculator, allowing general practices to calculate carbon generated from their non-clinical actions (See Sustainability, 2021).

Several challenges have been identified relating to the implementation and integration of these activities. A recent survey of general practice staff in England found organizational and systemic factors, such as difficulties engaging the wider practice team, alongside limited time, funding and higher-level support acted as barriers to decarbonization (Geddes et al., 2025). Additionally, it highlighted a lack of awareness around the resource materials available to support decarbonization (Geddes et al., 2025). Other research has identified effective leadership and staff engagement as crucial facilitators to the integration of decarbonization actions (Fehrer et al., 2023; Pavli et al., 2023; Wild et al., 2023; Nunes et al., 2025). Staff knowledge and awareness of climate change and its health impacts are also important (Boland and Temte, 2019; Müller et al., 2024; Nunes et al., 2025), and education and training on decarbonization is recognized to be essential for enhancing general practice staff competence (Guggenheim, 2016; Fehrer et al., 2023; Wild et al., 2023; Müller et al., 2024). While such interventions have been recommended to improve staff knowledge and skills, their implementation have not been widely evaluated (Fehrer et al., 2023; Wild et al., 2023; Müller et al., 2024).

Three key factors necessary for successful change in healthcare organizations include having the opportunity to influence change, valuing the change, and being prepared for the change (Nilsen et al., 2020). These are particularly relevant when considering strategies to encourage decarbonization in general practice, due to the numerous challenges around its implementation in this setting (Geddes et al., 2025; Nunes et al., 2025). Of specific relevance in driving action is the coalescing of ideas for change into a practice-level action plan (NHS Improvement, 2011). To aid their development, video interventions have been identified as valuable tools for delivering education to healthcare teams, and previous work in other contexts has demonstrated their effective use to enable change in knowledge, attitudes, skills and behaviours (Singh et al., 2015; Weakley et al., 2017; Morgado et al., 2024). Other advantages of video interventions include standardization of teaching (Keenan et al., 2021), increased accessibility, cost-effectiveness, and the ability for healthcare teams to view the material multiple times at their convenience, a factor particularly important in a setting with busy staff schedules and several competing demands (Pisani et al., 2021; Winiger et al., 2021; Morgado et al., 2024; Raja-Ismail et al., 2024).

Given this context, this study was designed to develop and evaluate the feasibility of an intervention to support practice team awareness-raising and decarbonization planning. The intervention comprised the presentation of an awareness-raising educational video to increase understanding of climate change and actions that can be undertaken in general practice settings, followed by a facilitated discussion focussed on action planning. The intended outcome was that general practice teams would plan the implementation of decarbonization actions in a structured, objective-focused green action plan (GAP).

### Aim and objectives

Aim: To describe the development, delivery, and outcome of an action-oriented intervention comprising an awareness-raising educational video and workshop designed to support general practice teams to identify and plan decarbonization actions in a GAP.

Objectives:

- To describe the development and delivery of the intervention to support the identification and planning of decarbonization actions in general practice.
- To evaluate workshop participants’ perceptions regarding the relevance and usefulness of the intervention in a range of general practice settings.
- To examine the impact of the intervention in supporting action planning through evaluation of the GAPs developed by practice teams.

## Materials and methods

The design of the intervention was guided by organizational change theory, specifically three factors for successful change in healthcare settings: opportunity to influence, valuing the change, and preparedness (Nilsen et al., 2020). We integrated video-design principles (Brame, 2016), and knowledge about barriers to decarbonization (Geddes et al., 2025; Nunes et al., 2025) to create an intervention in a format that could be implemented across general practices nationally: an awareness-raising educational video together with facilitator notes and supporting resources for practice-based workshop delivery.

### Video development methods

Video development involved pre-production, production, and post-production phases following a method used for clinical education films (Elliot et al., 2013; Farahani et al., 2021).

### Pre-production

When developing the educational video, we considered cognitive load, viewer engagement, and active learning to maximize its utility (Sweller, 2011; Brame, 2016; Farahani et al., 2021). Hence, we split the video into two segments that could be delivered during an online workshop, with each part followed by a facilitated discussion to reduce continuous viewing time and boost engagement and learning outcomes (Plass et al., 2012; Krumm et al., 2022).

The video was designed in sections, each ‘bookended’ by an introduction and summary slide to highlight key information, drawing on segmenting (the principle of dividing content into smaller units) and signalling (summarizing and highlighting key concepts) to aid information processing (Ibrahim et al., 2011; Brame, 2016). These techniques reduce learners’ self-reported mental effort (Mayer, 2009) and enhance knowledge acquisition (Mautone and Mayer, 2001; Ibrahim et al., 2011).

We drafted a storyboard to map content flow (Krumm et al., 2022), then iteratively developed a script and PowerPoint slides. We balanced audio and visual elements to avoid cognitive overload (Farahani et al., 2021; Krumm et al., 2022). Drafts were reviewed by the research team, members of the study’s Patient and Public Involvement (PPI) panel, and key external stakeholders in the field of sustainable general practice to refine relevance, clarity, and engagement.

We recruited four general practitioners (GPs) active in decarbonization to submit short, 1–2 minute ‘talking head’ clips, sharing practical examples relating to a key area of decarbonization. Participants filmed these independently and submitted them by email for incorporation into the main video. Talking heads have been shown to increase viewer satisfaction and engagement (Guo et al., 2014; Sondermann and Merkt, 2023).

### Production

We recorded narration over PowerPoint slides using Universal Capture Echo360, enabling in-built editing and easy video sharing via a digital link.

### Post-production

In this final phase, footage was edited using the Echo360 editing software. Editing focused on the insertion of the ‘talking head’ clips at the relevant points.

### Video content

The video was structured into four sections: 1. Climate change and health, 2. Net zero and primary care, 3. General resources to aid decarbonization in general practice, 4. Key areas for decarbonization in general practice. Section details are described in Supplementary file 1.

Section 1 detailed the rationale for decarbonization in general practice, outlining the impact of climate change on health, and conversely, the impact of healthcare on the climate.

Section 2 linked this to general practice, describing emissions hotspots from primary care and the NHS net zero ambition (NHS, 2020). These two sections were included to cover ‘valuing the change’, ensuring general practice teams had an understanding as to why decarbonization was necessary (Nilsen at al., 2020).

Section 3 and 4 were guided by previous research indicating a lack of knowledge around the decarbonization resource materials and initiatives feasible in general practice (Geddes et al., 2025), encompassing the principle of ‘having the opportunity to influence change’ (Nilsen et al., 2020). Section 3 summarized key resource materials available to GP teams to support decarbonization, and section 4 outlined six key areas for decarbonization action: asthma care, medicines use and waste, active travel, managing waste, energy use, and business services and procurement. Content for these two sections were identified through thorough scoping and stakeholder engagement to ensure a comprehensive overview and relevance to viewers.

The finalized video had a duration of 37 minutes and 27 seconds (13 minutes and 31 seconds for the first part, and 23 minutes and 56 seconds for the second part as shown to practices). The video can be freely viewed at: https://www.youtube.com/watch?v=1ZskhnTG3ok.

### Workshop design

The workshop was designed to deliver the educational video together with facilitated discussions to support the planning of decarbonization actions by general practice teams. Time pressures and capacity issues in general practice are well understood (André et al., 2022; Nunes et al., 2024; Geddes et al., 2025). Presenting the video during a workshop allowed for full, committed viewership from staff, with no expectations for reading or viewing materials beforehand. A supportive workplace culture that values sustainability has shown to be crucial for successful decarbonization (Pavli et al., 2023; Wild et al., 2023), and the convening of multidisciplinary practice staff in this workshop was intended to enable shared commitment to decarbonization and ownership of their subsequently developed GAP actions.

The workshop was designed to have a total duration of 90-minutes, a length deemed reasonable and feasible to deliver within a setting as busy as general practice. It combined the video screening with two facilitated discussions to translate learning into action planning (Table 1).

**Table 1.** Description of the structure and content of the workshop

Section	Description
Introduction	–Introductions –Workshop overview, expectations, and aims –Introducing the GAP template
Video, part 1 (see Supplementary file 1 for full description)	–Section 1- Climate change and health –Section 2- Net zero and primary care –Section 3- General resources to aid decarbonization in general practice
Facilitated discussion 1 (see Supplementary file 3 for full details of discussion prompts)	–Reflections on resource materials available to GP teams (Appendix 3)
Video, part 2 (see Supplementary file 1 for full description)	–Section 4- Key areas for decarbonization in general practice
Facilitated discussion 2 (see Supplementary file 3 for full details of discussion prompts)	–Reflections on previous experiences with decarbonization within the practice –Reflections on ideas for action –Action planning (Supplementary file 3)
Conclusion	–Next steps, developing the GAP –Conclusion and questions

There was a 10-minute discussion mid-way through the video and a 25-minute discussion at the end. The first facilitated discussion covered participants’ response to the resource materials to support decarbonization in general practice that had been presented in the video. The second facilitated discussion explored participants’ previous experience of undertaking decarbonization actions in their practice, potential areas for action, then prioritizing and planning how they will be enacted.

Facilitator briefing notes were informed by a combination of sociological and behavioural theories, Normalization Process Theory (NPT) (Murray et al., 2010) and the Theoretical Domains Framework (TDF) (Atkins et al., 2017), respectively. This was to enable the systemic identification of the cognitive, affective, and practice environmental determinants relevant to the planning of decarbonization actions within general practice and understanding the dynamic social and individual processes involved (Murray et al., 2010; Atkins et al., 2017). Prompts were iteratively refined following intervention delivery to the first three practices, which included moving prompt 6, relating to each practice’s prior experience of decarbonization, from the first, to the second facilitated discussion (Supplementary file 2).

Each workshop started with introductions between participants and the research team, and presentation of the aims and expectations of the workshop. Then, a GAP template was shared (Supplementary file 3); this had sections in which the practice team was expected to stipulate an area of activity, a designated lead, how they intend to undertake the action, a timeline for achievement, what success would look like, and any resources they intend to use to support their action (NHS Improvement, 2011). It was explained that following the workshop, practice teams would have two weeks to establish between three and five decarbonization actions to be undertaken in their practice over the subsequent 12 months; this short deadline was set to create momentum around the process.

Following the viewing of the video and the two facilitated discussions, a link to the video was shared, should members of the practice team want to watch particular sections in their own time, and next steps were summarized.

### Piloting the intervention

The intervention was piloted with a practice manager, GP trainee, and practice nurse across three different practices. Feedback on the intervention’s structure, content, and pacing led to minor adjustments before full rollout.

### Recruitment and sample

The intervention was delivered to twelve purposively sampled general practices from three diverse Integrated Care Boards (ICB) (subregional partnerships that bring together local health and care organizations) in England: four practices in each of Birmingham and Solihull, Coventry and Warwickshire, and South Yorkshire regions. Practices were recruited from those who registered interest through a survey aimed at understanding current levels of general practice staff interest in, and engagement with decarbonization initiatives (Geddes et al., 2025). Practices were selected to ensure maximal diversity across a variety of characteristics, including practice setting (urban/rural), list size, staff mix, population ethnicity, level of deprivation (based on the 2019 index of multiple deprivation (IMD) score, the official measure of deprivation in England), and current engagement with decarbonization. First contact was made through email, affirming participant interest, and practices were provided with information about the study and its requirements. Twelve practices were initially invited to participate; however, the early attrition of two practices necessitated the recruitment of replacement practices. All practices were financially remunerated for the time involved in their participation.

### Intervention delivery and evaluation

Workshops were delivered online via Microsoft Teams^TM^ from May-September 2024, at a time convenient to each practice. Facilitated discussions were recorded for separate qualitative analysis that will be reported in a future publication.

### GAP analysis

A conceptual content analysis was performed on the participating practices’ GAPs to categorize selected actions and link them to video content (White and Marsh, 2006; Sabharwal et al., 2016). This was performed by the lead author, and no inter-rater reliability was formally assessed. Coding categories were defined *a priori*, determined from the action area categories as described in the video presentation.

### Evaluation form

To assess workshop participants’ satisfaction and perception of the intervention, attendees were asked to compete a short, anonymous online survey hosted on the platform Qualtrics Provo (V7.24). The nine-item survey was developed by the study team and questions were not formally validated. It was distributed to attendees through a link shared using the Microsoft Teams^TM^ chat function, and through email following the intervention session (May–September 2024). Usefulness of the intervention was rated on a 5-point Likert scale. Other closed questions sought to elicit views on the video’s content, duration and quality. Two free-text items invited respondents to share key insights and provide feedback (Supplementary file 4).

Data were exported from Qualtrics and analysed in IBM SPSS (Version 29.0.1.0(171)). Descriptive statistics including absolute and relative frequencies were used to describe responses. Means, medians and response ranges were reported as appropriate. Free-text responses were thematically analysed. The coding framework was developed using an inductive approach, following a six-stage process as defined by Braun and Clarke (Braun and Clarke, 2006; Ziebland and McPherson, 2006; Turk et al., 2020; Khan et al., 2024). Coding was completed in Lumivero NVivo (Version 1.7.1 (1534)) and themes were identified independently by the lead author.

### Patient and Public Involvement (PPI)

Patients and public were involved in the conceptualization and design of the broader GPNET-0 Study. Two PPI panel members contributed to video design and reviewed workshop materials for clarity, ensuring patient perspectives shaped content and delivery.

## Results

### Practice demographics and workshop attendees

The participating general practices had a median list size of 7,400 (range: 1,500–18,900) and a median IMD decile of 7.4 (range: 1–10). The median number of workshop attendees was 5 (range: 4–7), comprising a diverse range of staff roles, including GP partners (16; 25.0%), practice mangers (10; 15.6%), reception staff (9; 14.1%), practice nurses (8; 12.5%), pharmacists (4; 6.3%), salaried GPs (2; 3.1%), and other clinical (5; 7.8%) and non-clinical staff (10; 15.6%). For a detailed profile of participating sites and workshop attendees, see Supplementary file 5.

### Action plan content analysis

All 12 case study practices returned their GAPs within two weeks of the workshop. Two of the first four returned GAPs included several sparsely detailed actions. We met with these practices and advised them to consolidate their plans by prioritizing 3–5 detailed actions, outlining the steps necessary for their completion; they were given an additional two weeks to refine their GAP. This prompted the development of example actions, unrelated to the topic area, integrated into the GAP template to guide practice teams’ decarbonization planning (Supplementary file 3).

Before describing the GAPs, it is important to reiterate that actions within them represent planning intentions, and not interventions already implemented. Practices described between three and eight decarbonization actions in their GAPs, with a median of 5. Actions spanned all six of the video-presented action areas. ‘Managing waste’ was the most frequent, present in 10 practice GAPs. List size and prior engagement with decarbonization were the only characteristics that related to the number of actions planned, with larger practices and those with higher prior engagement in decarbonization generally planning a higher number of actions. Table 2 summarizes areas identified for GAP actions.

**Table 2.** A summary of areas for action present in the returned practice GAPs

Area for action	Number of practices	Specific actions (number of practices)	Link to video
Managing waste	10	Encouraging domestic recycling (6) *e.g. Establishing recycling contracts with a waste provider; Clear signage for staff describing what can go in each waste stream*	Section 4- Managing waste
		Reduce unnecessary printer use (6) *e.g. Centralizing practice printing; Shifting to electronic communication methods with patients where letters have previously been sent*	Section 4- Managing waste
		Encouraging correct disposal of clinical waste (4) *e.g. Clear signage for staff describing what can go in each waste stream*	Not directly referenced in video
Medicines use and waste	9	Recycling blister packs (7) and inhalers (6) *e.g. Educating patients (through signage, texts, information on prescription reminders) about why and how to dispose of their blister packs and inhalers correctly; Providing blister pack and inhaler disposal services within their practice*	Section 4- Medicines use and waste
		Medication reviews to reduce unnecessary polypharmacy (4)	Section 4- Medicines use and waste
		Engage with the practice social prescriber (2)	Section 4- Medicines use and waste
		Recycling injectable pens (2) *e.g. Signposting patients to a free injectable pen recycling service*	Section 4- Medicines use and waste
Business services and procurement	9	Encourage appropriate resource use (4) *e.g. Signage and staff education around glove/apron use*	Section 4- Business services and procurement
		Order sustainable products (3) *e.g. Order reusable supplies (e.g. metal instead of plastic kidney dishes); Order products with packaging that can be more easily recycled*	Section 4- Business services and procurement
		Ordering practice supplies in bulk (3)	Section 4-Business services and procurement
		Incorporate reusable batteries (2)	Not directly referenced in video
		Transition to a greener supplier (1) *e.g. Use product suppliers with sustainability policies*	Section 4- Business services and procurement
Energy use	9	Encouraging mindful energy use (6) *e.g. Use of signage to encourage staff to switch off appliances when not in use*	Section 4- Energy use
		Installation of solar panels (3)	Section 4- Energy use
		Installing automated sensor lights (2)	Section 4- Energy use
		Switch to energy-efficient lighting (1)	Section 4- Energy use
		Switch to greener energy provider (1)	Section 4- Energy use
Asthma care	6	Review inhalers and switch to lower emission inhalers where appropriate (6)	Section 4- Asthma care
Active travel	5	Conducting a travel survey (3)	Section 4- Active travel
		Sign up to the government cycle to work scheme (3)	Section 4- Active travel
		Provide facilities to encourage active travel in the practice (2) *e.g. Installing secure bike storage*	Section 4- Active travel
		Facilitating car sharing (2) *e.g. Adjusting rotas to allow for staff to travel together*	Section 4- Active travel
		Sharing routes for active travel (1)	Section 4- Active travel

Other miscellaneous actions included installing a water butt and introducing meat-free days in one practice and declaring a climate emergency in another.

Seven practices’ GAPs specified resource materials presented in the video as being used to support their planned actions, a ‘high quality, low carbon’ asthma care toolkit (five practices), the Green Impact for Health toolkit (three practices), e-learning modules (two practices), a waste audit form (two practices), and an environmentally focussed inhaler patient decision-aid (two practices). Deprescribing toolkits appeared in two GAPs.

Across all 12 GAPs, each specified action was assigned to at least one lead individual. Seven practices had at least one action with two named leads. In one practice, a single individual led all actions. Practice managers were the most frequent action leads (20 actions), followed by other non-clinical staff (13 actions), practice nurses (12 actions), GP partners (11 actions) and reception staff (9 actions).

Generally, clinical actions were led by clinical staff, and non-clinical actions by non-clinical staff. Across the six practices with actions around asthma care, all had a practice nurse as lead, aside from one which had a pharmacist and a salaried GP as co-leads. No other action area had a uniform lead across GAPs.

Most GAPs were comprised of actions with a mix of both shorter and longer timeframes, however just under half of all actions were intended for implementation across the majority of the study’s 12-month duration (1–3m: 15 (22.7%); 4–6m: 12 (18.2%); 7–9m: 6 (9.1%); 10–12m: 29 (44.0%); no timeframe stipulated: 4 (6.1%)). Some actions were given a time frame of 12–months, with effort made to review action progress every few months.

### Evaluation

Thirty participants (46.9% of attendees) completed the intervention evaluation form. The mean number of responses per practice was 2.5, with a range of 1–4. The low response rate potentially introduces response bias as more engaged participants’ views may be overrepresented.

The intervention was found to be highly useful. On a scale of 1–5 (1 ‘not at all useful’ to 5 ‘very useful’), the workshop had a median rating of 5, with 28 (93.3%) respondents rating the overall usefulness of the workshop content as 4 or 5 (Table 3). The video had a median rating of 4, with 28 (93.3%) respondents ranking the video content overall as 4 or 5 (Table 3). The usefulness of the four sections of the video were rated similarly, each with a median rating of 4 or 5. All aspects of the intervention had at least one participant rating its usefulness as 3, and one respondent rated section 2 of the video as 2.

**Table 3.** Participant’s perception of the usefulness of the intervention on a scale of 1-5 (1 ‘not at all useful’ to 5 ‘very useful’)

	1	2	3	4	5	Median
The content of the workshop *n(%)*	0 (0.0%)	0 (0.0%)	2 (6.7%)	10 (33.3%)	18 (60.0%)	5
The content of the video *n(%)*	0 (0.0%)	0 (0.0%)	2 (6.7%)	16 (53.3%)	12 (40.0%)	4
Video, Section 1- Climate change and health *n(%)*	0 (0.0%)	0 (0.0%)	2 (6.7%)	18 (60.0%)	10 (33.3%)	4
Video, Section 2- Net zero and primary care *n(%)*	0 (0.0%)	1 (3.3%)	1 (3.3%)	15 (50.0%)	13 (43.3%)	4
Video, Section 3- General resources to aid decarbonization in general practice *n(%)*	0 (0.0%)	0 (0.0%)	3 (10.0%)	12 (40.0%)	15 (50.0%)	4.5
Video, Section 4- Key areas for decarbonization in general practice *n(%)*	0 (0.0%)	0 (0.0%)	1 (3.3%)	15 (50.0%)	14 (46.7%)	4

Perceptions of the intellectual level of the video varied, with 17 (56.7%) respondents deeming it to be of an ‘intermediate’ level, and 12 (40.0%) considering it to be ‘introductory’. Most (23; 76.7%) felt its length was appropriate; 7 (23.3%) found it too long. Visual and acoustic quality both had a median of 3, rated on a scale of 1–5 (1 being poor and 5 being excellent) (Table 4). Audio quality concerns (63.3% rated 3/5) highlight a technical limitation requiring immediate attention for future implementation.

**Table 4.** Participants’ perceptions of the audio and visual quality of the video on a scale of 1-5 (1 being poor and 5 being excellent)

	1	2	3	4	5
Quality of the video’s visuals *n(%)*	0 (0.0%)	1 (3.3%)	15 (50.0%)	11 (36.7%)	3 (10.0%)
Quality of the video’s acoustics *n(%)*	0 (0.0%)	2 (6.7%)	19 (63.3%)	7 (23.3%)	2 (6.7%)

On a scale of 1–5 (1 being unlikely, and 5 being very likely), respondents were asked to rate the likelihood of sharing the video with colleagues in the practice. 24 (80%) respondents reported 4 or 5, with a median rating of 4.

### Free-text results

The first free-text question asked respondents to share key things learnt from the video and workshop. Twenty-five (83.3%) respondents replied, generating themes of i) *information about the topic area*, ii) *resource materials*, and iii) *ideas for action*. The second asked for improvement suggestions. Seventeen (56.7%) responded, citing: i) *time to consolidate learning from the video within the workshop*, ii) *the video’s audio quality*, and iii) *a shortened duration of the video and workshop*. Table 5 provides quotes illustrative of the themes.

**Table 5.** Quotes illustrative of themes identified from free-text items of the workshop and video evaluation

Question	Theme	Illustrative quotes (with respondent codes, R*number* is the respondent ID, and the letter signifies the practice code)
*What key things did you learn from the video presentation/workshop?*	Topic area	–‘how the NHS and primary care (is) responsible for carbon emissions in Britain’- R.25.B –‘the main causes of pollution (in general practice)’- R.17.I
	Decarbonization resource materials	–‘useful resources and toolkits’- R.04.A –‘lots of resources- perhaps too many’- R.19.K
	Ideas for action	–‘access to ideas to target’- R.24.C –‘Good ideas which we can certainly look at implementing within the practice’- R.12.G
*How could the video/workshop be improved?*	Time to consolidate learning within the workshop	–‘The video or resources could have been provided before the meeting as background, and the meeting used for some creative thinking’- R.19.K –‘I think we could watch this in our own time before a meeting and then have time to reflect then come together to consider our action plans’- R.24
	Audio quality	–‘Sound was an issue at times’- R.29.D –The GP videos at the end some of the vocal was out of sync’- R.01.L
	Shortened duration	–‘Shorter time if possible’- R.15.E –‘I found it very frustrating to be sat watching a very long video’- R.19.K

## Discussion

In this study, we have described the development and delivery of an action-oriented intervention to overcome barriers to decarbonization in general practice. Our low-cost, scalable approach supported practice teams to identify and plan decarbonization actions. Overall, the intervention was well received and rated as useful for action planning, although some participants identified scope for shortening its duration, and improving audiovisual quality. Practice teams generated GAPs that identified numerous decarbonization actions to be undertaken over the subsequent 12 months, each with a designated lead, specified outcomes and a timescale.

### Strengths and limitations

A strength of this work was the purposive sampling based on varied practice characteristics and levels of prior engagement with decarbonization, however, the small sample, 12 practices spanning 3 ICBs, limits generalizability to broader NHS contexts. Practices volunteered to take part in the study and were remunerated for their time, this selection bias potentially meaning that these practices were more motivated than others to enact change. Due to the busy nature of general practice, some found establishing a time to hold the workshop to be a challenge. Outside of study conditions, some practices may struggle to ringfence time to hold a workshop of this nature in the absence of financial incentives. Furthermore, the absence of a control group precludes causal inferences about intervention effectiveness.

The short timeframe between the intervention, the evaluation form and GAP development means that this study could not assess the actual implementation of the actions as developed within the GAP. This is beyond the scope of this study and will be reported in a future publication exploring the longitudinal implementation of GAP actions.

Another strength of this work was its grounding in theory, including principles around organizational change (Nilsen et al., 2020), and video-design to maximize viewer engagement, satisfaction, and knowledge acquisition (Guo et al., 2014). The development approach taken was low-cost and designed to succinctly cover a broad range of issues, introducing practices to a range of potential action areas and resource materials to support decarbonization, most of which they were unaware of (Geddes et al., 2025). The structure of the workshop permitted active discussion amongst team members and the opportunity to reflect and inform the development of their GAP. The intervention was designed for multidisciplinary practice staff, ensuring representation across numerous areas of a practice and broad staff engagement, previously identified as a facilitator to successful decarbonization (Fehrer et al., 2023; Pavli et al., 2023; Wild et al., 2023). The process of working together to develop a practice GAP encompassed ‘having the opportunity to influence the change’ and ‘being prepared for the change’, so enabling shared ownership and commitment to delivering the planned actions GAP (Neilsen et al., 2020).

A limitation of the video was its visual and audio quality, which were in part constrained by limited funding for video production and reliance on streamed web conferencing. It is possible that this may have affected engagement and learning outcomes, however the extent of this cannot be determined. Additionally, our sample of case study sites were selected to cover varied levels of engagement with decarbonization, and with that, we aimed to develop a video that presented a more rudimentary, comprehensive overview of decarbonization within this setting. This may have resonated less with practices already engaged in decarbonization. Further benefit may have been attained from videos tailored more specifically to GP teams’ level of knowledge and current engagement. Moreover, the field of decarbonization in general practice is fast evolving, and with it, the availability of resources to support and inform general practice staff to take action. The video needs to be regularly updated to ensure its relevance to current resource materials available to GP teams.

Finally, the evaluation form was designed to be brief to encourage uptake and completion. As a result, the questions were limited in scope and had a tick-box format and were not formally validated. Despite this, there was only a moderate level of response (46.9%), which potentially introduces response bias as more engaged participants’ views may be overrepresented, limiting the generalizability of the findings. Additionally, self-reported satisfaction data, completed immediately following the intervention, may have introduced social desirability bias. From the data collected, we were unable to identify detailed patterns within and across responses.

### How this relates to other research

Recommendations for video duration are contested, and there remains a lack of information around optimal video duration for healthcare professionals; however, between 5-20 minutes is deemed appropriate for educational videos with healthcare students (Dong and Goh, 2014; Guo et al., 2014; Krumm et al., 2022). The total length of our video exceeded this to ensure a comprehensive overview of the topic; however, this may have been at the detriment of participant attention, despite attempts to break the video up by displaying it in two parts (Guo et al., 2014).

Mapping GAP actions onto the action areas presented within the video, although deductive, permitted for a useful categorization of actions, with most actions mapping onto the video’s categories. The video was designed to detail a full spectrum of decarbonization actions feasible within general practice and was informed by the resources available at the time of development, further justifying this approach. The most frequently planned area for action was waste management (10 practice GAPs). Medicines use and waste, business services and procurement, and energy use actions were each present in nine practice GAPs, and asthma care and active travel were the least frequently selected action areas (in six and five practice GAPs respectively). This is in-line with other research which identified areas around waste management and energy usage as being most widely adopted, and actions around prescribing and active travel being less frequently enacted despite their significant contribution to a practice’s carbon footprint (Tennisson et al., 2021; Nicolet et al., 2022; Woodcock et al., 2022; Pavli et al., 2023). It has been posited that this may be due to a lack of staff knowledge regarding the impact of decarbonization actions (Pavli et al., 2023), however this was described within section 2 of the video presentation, so participants in this study would have had an awareness of emissions hotspots and their respective mitigative actions. Recycling and energy use are both domains of action commonly practiced domestically in the UK, which may have influenced their uptake. These are also both categories of action that are ‘visible’ to the practice team, and despite their relatively lesser impact, these ‘visible’ actions can support awareness raising around the issue of environmental sustainability within a practice, further stimulating staff to consider actions with a greater impact (Hale and Bell, 2023; Hale and McKenzie, 2024). It is also important to acknowledge external factors, such as practice location and availability of public transport, that may impact the feasibility of active travel (Woodcock et al., 2022).

In 2021, the Impact and Investment Fund (IIF) financially incentivized targets to encourage general practices in England to reduce the environmental impact of inhaler prescribing, and other incentives have existed at a local level to support this (NHS England, 2021). In our recent survey, reducing the environmental impact of inhaler prescribing emerged as an action most practices had reported undertaking (Geddes et al., 2025). This may further explain the lesser engagement compared to other action areas.

GAP action leads spanned individuals in both clinical and non-clinical roles. Other research has shown that a workplace culture that values sustainability can facilitate decarbonization initiatives (Pavli et al., 2023; Wild et al., 2023) but engaging the wider practice team can be a challenge (Geddes et al., 2025). The workshop design intended to overcome these challenges by ensuring the presence of multidisciplinary practice staff, and the varied roles of GAP action leads could enable the wider engagement necessary for successful implementation. Practices with a larger patient list size and higher prior engagement with decarbonization were likely to plan more actions in their GAP, potentially due to their increased staffing capacity and familiarity with the topic area.

### Learning points

This study demonstrates that a combined workshop-video intervention to support the planning of decarbonization actions in general practice settings is both feasible and acceptable. It was successful in supporting practices to develop GAPs, with the planned actions reflecting video content and recommended resources.

Nearly a quarter of respondents deemed the video to be too long. To overcome this, numerous, shorter video modules could be developed, and this would also address the previously described limitation of the ‘one size fits all’ video approach. It would enable practice teams to select and view content relevant to their specific knowledge level and practice needs. Segmenting video content in this way and permitting viewers to select the content they engage with can increase engagement, reduce cognitive load, and improve knowledge retention (Dong and Goh, 2014; Guo et al., 2014; Mayer, 2009; Brame, 2016).

An alternative study design could have utilized a control group to more effectively assess the effectiveness of the video method. The sample of general practices could have been split across two groups, ensuring similar characteristic spread, with one group being delivered the intervention as described, viewing the video presentation during a workshop session, and another having a workshop session with the video’s information presented in written form. Measures of workshop satisfaction and utility through the evaluation form and data from the resulting GAPs could be evaluated to provide further insights into the effectiveness of the video method.

### Implications

Our findings confirm the intervention’s feasibility and acceptability across diverse practice settings. By describing and reflecting upon these, we provide an opportunity for individuals to use these materials to support the planning and initiation of decarbonization actions in their own general practice settings. All materials, including the video (https://www.youtube.com/watch?v=1ZskhnTG3ok ), the facilitator notes (Supplementary file 2) and GAP template (Supplementary file 3) have been made publicly available to enable wider adoption.

Most intervention elements seem to be transferable across diverse practice settings, given that there was no discernible disparity in GAP outcomes across practices with differing characteristics. There are, however, ICB-level disparities in resources and funding available for decarbonization strategies; an example being the presence of sustainability fellows in Coventry and Warwickshire ICB, funded to support decarbonization in general practices in the area. These regional disparities mean that a generalized video would not necessarily be able to address all opportunities for decarbonization at a regional or local level.

It is important, also, to acknowledge that intentions alone may be insufficient to determine behaviour, dubbed the intention-behaviour gap (Sheeran and Webb, 2016). Resultingly, this study cannot assess GAP implementation, however a longitudinal evaluation of the intervention is currently underway (Nunes et al., 2024). This will enable understanding of the long-term effect that an intervention of this type can have, whilst identifying broader factors that influence the implementation of practice decarbonization actions.

## Conclusion

This paper has described the development, delivery, and outcome of an educational video-workshop intervention that facilitated multidisciplinary general practice teams to identify and plan decarbonization actions, highlighting the feasibility and acceptability of this intervention. This pilot intervention successfully engaged multidisciplinary teams in developing structured GAPs, however, without follow-up data on actual implementation, longer-term effectiveness in achieving decarbonization outcomes remains unknown. While knowledge and awareness barriers can be addressed through such interventions, organizational and systemic barriers including limited time, funding, and higher-level support also need to be considered (Geddes et al., 2025). It is essential that these barriers to decarbonization are addressed for an intervention of this type to become widely used.

## Supporting information

Geddes et al. supplementary material 1 Geddes et al. supplementary material

Geddes et al. supplementary material 2 Geddes et al. supplementary material

Geddes et al. supplementary material 3 Geddes et al. supplementary material

Geddes et al. supplementary material 4 Geddes et al. supplementary material

Geddes et al. supplementary material 5 Geddes et al. supplementary material

## Data Availability

Research data for this article includes data from the evaluation form and the GAP content. All information requests should be submitted to the corresponding author for consideration.

## Supplementary material

The supplementary material for this article can be found at https://doi.org/10.1017/S1463423626101145

## Equality, Diversity and Inclusion (EDI) and sustainability

- The intervention was delivered over online video call at a time convenient to the practice team to ensure accessible participation. We encouraged the inclusion of practice staff across a across a variety of roles.
- Closed captions were made available on the video.
- The intervention was delivered remotely to ensure accessible participation and to reduce greenhouse gas emissions associated with travel to practice sites.
