# Supporting general practices to develop green action plans to reduce carbon emissions: development and evaluation of the feasibility of a workshop-based intervention – CORRIGENDUM

**DOI:** 10.1017/S1463423626101388

**Published:** 2026-06-02

**Authors:** Olivia Geddes, Helen Twohig, Abi Eccles, Helen Atherton, Frederik Dahlmann, Florence Karaba, Ana Raquel Nunes, Rachel Spencer, Nicky Thomas, Jeremy Dale

On submission, the author Ana Raquel Nunes was accidentally noted as Anna Raquel Nunes. The correct spelling has now been updated. No other aspects of the article or the author’s linked ORCID have been altered. The authors apologise for this error.
