# Supplementary material for: Supporting general practices to develop green action plans to reduce carbon emissions: development and evaluation of the feasibility of a workshop-based intervention
Source: Prim Health Care Res Dev. 2026 Mar 27;27:e40. doi: 10.1017/S1463423626101145 (PMC13080534; doi:10.1017/S1463423626101145)
Supplement: Geddes et al. supplementary material 1 — Geddes et al. supplementary material [file S1463423626101145sup001.docx]

*Supplementary file 1- Description of the video’s structure and content*

| Section | Description |
| --- | --- |
| Introduction | - A title and contents slide, with a narration introducing viewers to the video and summarising what will be covered. |
| Section 1- Climate change and health | - The first section covers the rationale for decarbonisation in general practice, detailing the links between the changing climate and health, including the impact that the climate has on health, and the impact healthcare has on the climate. - It also introduces the NHS net zero ambition. |
| Section 2- Net zero and primary care | - The second section details the environmental impact of primary care, including emissions hotspots across the primary care estate. - It then introduces two frameworks to be applied to decarbonisation, the ‘reduce, reuse, and replace’ hierarchy, alongside the principles of sustainable healthcare, as developed by the Centre for Sustainable Healthcare (<https://sustainablehealthcare.org.uk/>) (CSH., 2008). |
| Section 3- General resources to aid decarbonisation in general practice | - This section gives an overview of general resources designed to support decarbonisation in general practice. It explains what each resource does, how they can be used, and how they can be accessed. The following resources were included:   - RCGP Net Zero e-learning hub courses(<https://elearning.rcgp.org.uk/course/view.php?id=650&dm_t=0,0,0,0,0>) (RCGP., 2023)   - RCGP ‘Decarbonising General Practice Guide’ (<https://elearning.rcgp.org.uk/course/view.php?id=650&dm_t=0,0,0,0,0>.)(RCGP., 2022)   - Green Impact for Health Toolkit [(https://toolkit.sos-uk.org/greenimpact/giforhealth/login](%20(https://toolkit.sos-uk.org/greenimpact/giforhealth/login)) (Green Impact., 2015)   - Greener Practice Network and website (<https://greenerpractice.co.uk>)(Greener Practice., 2017)   - Centre for Sustainable Healthcare website (<https://sustainablehealthcare.org.uk/>) (CSH., 2008)   - Cheshire and Merseyside ICB 10-point Green Plan for Primary Care (NHS Cheshire and Merseyside., 2023) (<https://www.cheshireandmerseyside.nhs.uk/media/hahluufe/10-point-plan-for-primary-care.pdf>)   - Cornwall Greener Practice 15-point Climate and Nature Action Plan (Volunteer Cornwall., 2023) (<https://www.healthandclimateresilience.net/_files/ugd/4f7a58_68d8aae482be4e378d51a347dab3fbe0.pdf>)   - High Quality and Low Carbon Asthma Care Toolkit (<https://www.greenerpractice.co.uk/high-quality-and-low-carbon-asthma-care/>) (Greener Practice., 2022) |
| Introduction to the second part- | - The narrator describes the contents covered in the second part of the video. |
| Section 4- Key areas for decarbonisation in general practice | - The final section outlines 6 key areas for decarbonisation action, providing examples of actions that can be undertaken and specific resources that can be used to support them. The key areas included are:   - Asthma care   - Medicines use and waste   - Active travel   - Managing waste   - Energy use   - Business services and procurement - Short clips of GP staff from across England were interspersed between each action area, describing an action they had undertaken, and the impact this has had on the practice. |
| Outro- | - The final slide thanks the viewer for watching and encourages them to think about decarbonisation actions they could undertake in their practice. |
