# Supplementary material for: Supporting general practices to develop green action plans to reduce carbon emissions: development and evaluation of the feasibility of a workshop-based intervention
Source: Prim Health Care Res Dev. 2026 Mar 27;27:e40. doi: 10.1017/S1463423626101145 (PMC13080534; doi:10.1017/S1463423626101145)
Supplement: Geddes et al. supplementary material 2 — Geddes et al. supplementary material [file S1463423626101145sup002.docx]

*Supplementary file 2- Facilitated discussion prompts*

| **Facilitated Discussion 1-** |
| --- |
| 1. Have you heard of, or used, any of the resources presented in the video? 2. What do you think of them? 3. Are any particularly helpful/what was good about them? 4. Were there any particular challenges you found when using them? 5. How easy were they to incorporate into usual working of the practice? 6. Were there any presented that you haven’t used that look particularly appealing? 7. How easy do you think they would be to incorporate into the usual working of the practice? 8. Do you foresee any issues with using them? 9. Were there any there that you thought were unappealing or wouldn’t work for your practice, why? 10. Some of the resources come with awards, the GIFHt for example, you can get RAG, is this sort of reward-based system appealing to you? 11. There were a few resources that talked about local networks, do they appeal to you? |
| **Facilitated Discussion 2-** |
| 1. We would like to start the discussion by getting an idea about what your practice is doing currently, if anything, in efforts to decarbonise? 2. Briefly describe what you have been doing. 3. What has worked well? 4. What challenges have you faced? |
| 1. Get your initial reflections on the ideas presented in the video. 2. Which of these actions do you see being able to take forward within your practice? Why? 3. Are there any that are less appealing? Why? 4. What kind of impact do you think these actions would have? Positive impacts? Negative impacts?  (disruption, financial, staff or patient experience) 5. What kind of challenges might you face? |
| 1. Now, we would like to discuss the best ways to enact these changes. If you would like to make notes during this part, it could be helpful as you complete the GAP over the next couple of weeks. Let’s start by considering one or two actions identified. (Any preference?) 2. How will you enact these changes? What actions would you take? 3. Who within your team needs to be involved? What roles would people play? How will you ensure staff members are engaged? 4. Who will be responsible for overall coordination and leadership over the 12-months? 5. What resources and support do you think is needed to make these changes? How will you access these? 6. Can you think of ways to overcome any of the challenges we discussed earlier? Are there opportunities for your PPG to be involved? How? |
| 1. For the final part of this workshop, we would like to talk about the ways in which you will monitor and maintain these actions? 2. How will you monitor the actions you are carrying out? 3. How will you assess what is working well and what is not working well? |
