# Supplementary material for: Supporting general practices to develop green action plans to reduce carbon emissions: development and evaluation of the feasibility of a workshop-based intervention
Source: Prim Health Care Res Dev. 2026 Mar 27;27:e40. doi: 10.1017/S1463423626101145 (PMC13080534; doi:10.1017/S1463423626101145)
Supplement: Geddes et al. supplementary material 3 — Geddes et al. supplementary material [file S1463423626101145sup003.docx]

*Supplementary file 3- Green Action Plan (GAP) Template*

**Green Action Plan Template**

| Practice name: | Date: |
| --- | --- |

| **Decarbonisation Area of Activity** | **Lead individual(s)** | **Agreed Action(s)** | **When will this be achieved by?** | **What resources will you use, and what will success look like?** |
| --- | --- | --- | --- | --- |
| *Example 1.*  Reduction in meat eaten at home |  | 1. Discuss with family to explain reasons and ensure everyone is in agreement with the aim 2. Calculate current amount spent on meat per weekly shop 3. Look at recipes and identify at least 5 meat free meals that everyone is happy with 4. Try one meat free meal per week initially 5. Increase number of meat free meals per week over time   Calculate amount spent on meat per weekly shop to assess change | Plan in place by July 24  Start weekly meat free meals in August  Increase over subsequent 3 months  Assess progress in November | Vegetarian cook books / recipe web sites  <https://bslm.org.uk/the-planetary-health-diet-and-community-food-growing-for-health/>  Outcomes:  -number of meat free meals per week during november  -reduction in spend on meat during weekly shop  - overall change in spend on weekly shop (though may be affected by other factors) |
| *Example 2.*  HRT prescribing – ensuring that people are on the correct dose of progesterone |  | 1. Review latest guidance on dose of progesterone needed for HRT with high dose oestrogen component (British Menopause Society) 2. Write brief guideline for practice team and template letters for patients 3. Ask GP registrars to carry this out for their QI project 4. Search for all those prescribed transdermal oestrogens 5. Virtual review of each person’s notes 6. If not on adequate progesterone, change prescription and send template letter 7. If on cyclical preparation when could be changed to continuous, send letter and make note for this to be discussed at next med r/v. 8. Record data on actions taken. 9. Present findings at practice meeting   Re-audit 6 months later | Planning stage by August 24  GP registrars to work on it Sep-Nov  Present to practice meeting in Dec  Re-audit June 25 | <https://thebms.org.uk/publications/bms-joint-guidelines/management-of-unscheduled-bleeding-on-hormone-replacement-therapy-hrt/>  100% of those on high dose oestrogen for HRT will have adequate progesterone component at time of re-audit |
|  |  |  |  |  |
|  |  |  |  |  |
|  |  |  |  |  |
|  |  |  |  |  |
|  |  |  |  |  |

**Areas to consider and the resources available to support your actions:**

| **General Resources**  (5:07) | **Green Inhaler Prescribing**  (15:13) | **Reducing Medicines use and Waste**  (19:15) | **Encouraging Active Travel**  (23:07) | **Managing Waste**  (27:16) | **Energy Use**  (29:43) | **Business Services and Procurement**  (33:10) |
| --- | --- | --- | --- | --- | --- | --- |
| - [Carbon Literacy for Healthcare](https://carbonliteracy.com/healthcare/) - [RCGP Net Zero e-Learning Hub](https://elearning.rcgp.org.uk/course/view.php?id=650) - [Green Impact for Health toolkit](https://toolkit.sos-uk.org/greenimpact/giforhealth/login) - Greener Practice - [Website](https://www.greenerpractice.co.uk/) - [Local Group](https://www.greenerpractice.co.uk/join-our-network/local-groups/birmingham-and-solihull/) - [Centre for Sustainable Healthcare](https://sustainablehealthcare.org.uk/) - Primary Care Green Action Plan - [Cheshire and Merseyside](https://www.cheshireandmerseyside.nhs.uk/media/hahluufe/10-point-plan-for-primary-care.pdf) - [Southwest](https://www.healthandclimateresilience.net/s-projects-basic)   **Monitoring**   - [OpenPrescribing](https://openprescribing.net/) | - [High Quality and Low Carbon Asthma Care Toolkit](https://www.greenerpractice.co.uk/high-quality-and-low-carbon-asthma-care/)- Greener Practice   **Patient-related**   - [NICE Asthma inhalers and climate change](https://www.nice.org.uk/guidance/ng80/resources/inhalers-for-asthma-patient-decision-aid-pdf-6727144573)- patient decision aid - [Green Inhaler](https://greeninhaler.org/)- Patient facing website | - [BMJ Too Much Medicine Initiative](https://www.bmj.com/too-much-medicine) - [STOPP/START tool](https://link.springer.com/article/10.1007/s41999-023-00777-y) - [RCGP TARGET antibiotic toolkit](https://elearning.rcgp.org.uk/course/view.php?id=553) - [Anticholinergic burden calculator](https://www.acbcalc.com/) - [‘Show me your meds’ project](https://pharmaceutical-journal.com/article/opinion/show-me-your-meds-please-the-impact-of-home-based-medicines-assessments)   **Social Prescribing:**   - [Ramblers](https://www.ramblers.org.uk/go-walking/wellbeing-walks) - [British Nordic Walking](https://britishnordicwalking.org.uk/pages/about-nordic-walking) - [The Wildlife Trust](https://www.wildlifetrusts.org/nature-health-and-wild-wellbeing) | - [Love to ride](https://www.lovetoride.net/uk) - [Bikability](https://www.bikeability.org.uk/)- cycle training - [Letsride](https://www.letsride.co.uk/) - [Cycle to Work Scheme](https://www.gov.uk/government/publications/cycle-to-work-scheme-implementation-guidance) | - [SeeSustainability](https://seesustainability.co.uk/) Waste Audit Forms - [Medical room waste](https://img1.wsimg.com/blobby/go/15947d70-9404-4d54-921d-79cf246e9dc0/downloads/Medical%20waste%20audit%20form.pdf?ver=1714658419959) - Office [room waste](https://img1.wsimg.com/blobby/go/15947d70-9404-4d54-921d-79cf246e9dc0/downloads/Waste%20audit%20form.pdf?ver=1714658419959) | - [Guidance on conducting an independent energy audit](https://www.sseenergysolutions.co.uk/small-business-sustainability/business-energy-audits)- SSE Energy | **Business services:**   - [Green Directory](https://www.greendirectory.co.uk/)- UK’s Green Service Directory   **Procurement:**   - [Warp It](https://www.warp-it.co.uk/)- Loan, give away, or get second hand office equipment and consumables - [Seal Medical](https://www.sealmedical.com/)- reconditioned medical equipment - [Revolution zero](https://www.bing.com/search?q=revolution+zero&cvid=b66dd480766a4c9b8bfd04986ff6837a&gs_lcrp=EgZjaHJvbWUyBggAEEUYOTIGCAEQRRg8MggIAhDpBxj8VdIBCDIzODhqMGo0qAIIsAIB&FORM=ANAB01&PC=U531)- reusable IIR type face masks - [‘Gloves off’ campaign](https://www.england.nhs.uk/atlas_case_study/the-gloves-are-off-campaign/) |

GPNET-0 Study Workshop Video: <https://www.youtube.com/watch?v=1ZskhnTG3ok>

- **Section 1** (Climate Change and Health)- 0:00
- **Section 2** (Net Zero and Primary Care)- 3:13
- **Section 3** (General Resources to Aid Decarbonisation in General Practice)- 5:07
- **Section 4** (Key Areas for Decarbonisation in General Practice)- 13:32. See above for area specific timings.
