# Supplementary material for: Supporting general practices to develop green action plans to reduce carbon emissions: development and evaluation of the feasibility of a workshop-based intervention
Source: Prim Health Care Res Dev. 2026 Mar 27;27:e40. doi: 10.1017/S1463423626101145 (PMC13080534; doi:10.1017/S1463423626101145)
Supplement: Geddes et al. supplementary material 4 — Geddes et al. supplementary material [file S1463423626101145sup004.docx]

*Supplementary file 4- Evaluation form*

GPNET-0 Workshop Feedback Form

**Thank you for attending the GPNET-0 Study baseline workshop**

Your feedback on the workshop, including the video presentation, is critical to ensure we are meeting your practice's decarbonisation needs. We would really appreciate you taking a few minutes now to share your opinions with us. This information will be anonymised and used as part of our research.

Q1 Name of practice

________________________________________________________________

Q2 In the context of your practice's intention to reduce its carbon emissions, on a scale of 1-5 (1 'not at all useful' to 5 'very useful') how did you find...

|  | 1 | 2 | 3 | 4 | 5 |
| --- | --- | --- | --- | --- | --- |
| The content of the workshop overall |  |  |  |  |  |
| The content of the video presentation overall |  |  |  |  |  |
| Section 1 of the video- Climate Change and Health |  |  |  |  |  |
| Section 2 of the video- Net Zero and Primary Care |  |  |  |  |  |
| Section 3 of the video- General Resources to Aid Decarbonisation in General Practice |  |  |  |  |  |
| Section 4 of the video- Key Areas for Decarbonisation in General Practice |  |  |  |  |  |

Q3 In your opinion, what level of interest and involvement in decarbonisation would you consider the workshop and video presentation to have been pitched at.

|  | Introductory | Intermediate | Advanced |
| --- | --- | --- | --- |
| Workshop |  |  |  |
| Video presentation |  |  |  |

Q4 Given the topic and its purpose, was the length of the video presentation...

- Too short
- The right length
- Too long

Q5 Please rate the quality of the following from the video presentation...

|  | Poor | Fair | Good | Very Good | Excellent |
| --- | --- | --- | --- | --- | --- |
| Visuals |  |  |  |  |  |
| Acoustics |  |  |  |  |  |

Q6 On a scale of 1-5 (1 being unlikely, and 5 being very likely) how inclined would you be to...

|  | 1 | 2 | 3 | 4 | 5 |
| --- | --- | --- | --- | --- | --- |
| Recommend the workshop video to a colleague in your practice? |  |  |  |  |  |

Q7 What key thing(s) did you learn from...

- i. the video presentation

__________________________________________________

- ii. the workshop as a whole __________________________________________________

Q8 Is there anything that the workshop and video presentation did not touch on that would be useful to help you plan and undertake decarbonisation initiatives at your practice?

- Yes (add details) __________________________________________________
- No

Q9 How could the video/workshop be improved?

________________________________________________________________

________________________________________________________________

________________________________________________________________

________________________________________________________________

________________________________________________________________
