# Supplementary material for: Supporting general practices to develop green action plans to reduce carbon emissions: development and evaluation of the feasibility of a workshop-based intervention
Source: Prim Health Care Res Dev. 2026 Mar 27;27:e40. doi: 10.1017/S1463423626101145 (PMC13080534; doi:10.1017/S1463423626101145)
Supplement: Geddes et al. supplementary material 5 — Geddes et al. supplementary material [file S1463423626101145sup005.docx]

*Supplementary file 5- Profile of participating general practice case study sites*

| Practice | ICB | List size | IMD decile _a_ | Urban/Rural | Number of workshop attendees | Workshop attendee role _b_ |
| --- | --- | --- | --- | --- | --- | --- |
| A | Birmingham and Solihull | 1,500 | 1 | Urban | 6 | - PM - 2 GPp - PN - 2 R |
| B | Birmingham and Solihull | 18,900 | 2 | Urban | 5 | - GPp - 2 PN - R - Oc |
| C | Birmingham and Solihull | 5,500 | 10 | Urban | 7 | - PM - 3 GPp - R - Ph - Oc |
| D | Birmingham and Solihull | 5,600 | 1 | Urban | 4 | - PM - GPp - PN - O |
| E | Coventry and Warwickshire | 13,800 | 8 | Rural | 6 | - GPp - PN - R - Ph - 2 O |
| F | Coventry and Warwickshire | 5,300 | 8 | Rural | 4 | - PM - GPp - R - O |
| G | Coventry and Warwickshire | 7,900 | 8 | Rural | 4 | - GPp - R - Oc - O |
| H | Coventry and Warwickshire | 5,800 | 10 | Rural | 6 | - 2 PM - GPp - Oc - 2 O |
| I | South Yorkshire | 10,500 | 9 | Urban | 7 | - PM - GPp - GPs - PN - R - Ph - O |
| J | South Yorkshire | 13,800 | 2 | Urban | 5 | - PM - GPp - Ph - Oc - O |
| K | South Yorkshire | 8,300 | 4 | Urban | 5 | - PM - 2 GPp - PN - O |
| L | South Yorkshire | 6,900 | 7 | Urban | 5 | - PM - GPp - GPs - PN - R |
| _a_ *Measured by the index of multiple deprivation score at practice level (2019)*  _b_ *Abbreviations for GP staff role: PM- Practice Manager, GPp- GP Partner, GPs- Salaried GP, PN- Practice Nurse, R- Receptionist, Ph- Pharmacist, Oc- Other (clinical), O- Other (non-clinical)* | | | | | | |
